# Supplementary material for: Using domain knowledge for robust and generalizable deep learning-based CT-free PET attenuation and scatter correction
Source: Nat Commun. 2022 Oct 6;13:5882. doi: 10.1038/s41467-022-33562-9 (PMC9537165; doi:10.1038/s41467-022-33562-9)
Supplement: Supplementary file 1 — Supplementary material [file 41467_2022_33562_MOESM1_ESM.pdf]

SUPPLEMENTARY MATERIAL

Using domain knowledge for robust and generalizable deep learning-based CT-free PET attenuation and scatter correction

Methods

Decomposition-based DL. As shown in Figure 5, we first calculated the anatomy-dependent correction map (ADCM) based on non-attenuation and non-scatter corrected image ($I^{NASC-PET}$) and corrected PET ($I^{ASC-PET}$), which is a voxelwise ratio map (I^{ADCM}).

In order to preserve more spatial information, which is most essential for the task of attenuation and scatter correction, we zero-padded and downsampled the NASC-PET and ADCM to size of 112 ×112 ×112 from 440 ×440 ×448. Min-max normalization was applied to the entire dataset. To be noted that, when testing on external scanners, images are first resampled to the same voxel spacing as training data (1.65×1.65×2 mm), and then zero-padded and resized to 112 ×112 ×112.

Deep Neural Network design. The goal of the generator is to be able to approximate the corresponding ratio map for a given non-attenuation and non-scatter corrected images, while the discriminator aims to distinguish between the synthesized ratio map and the real input.

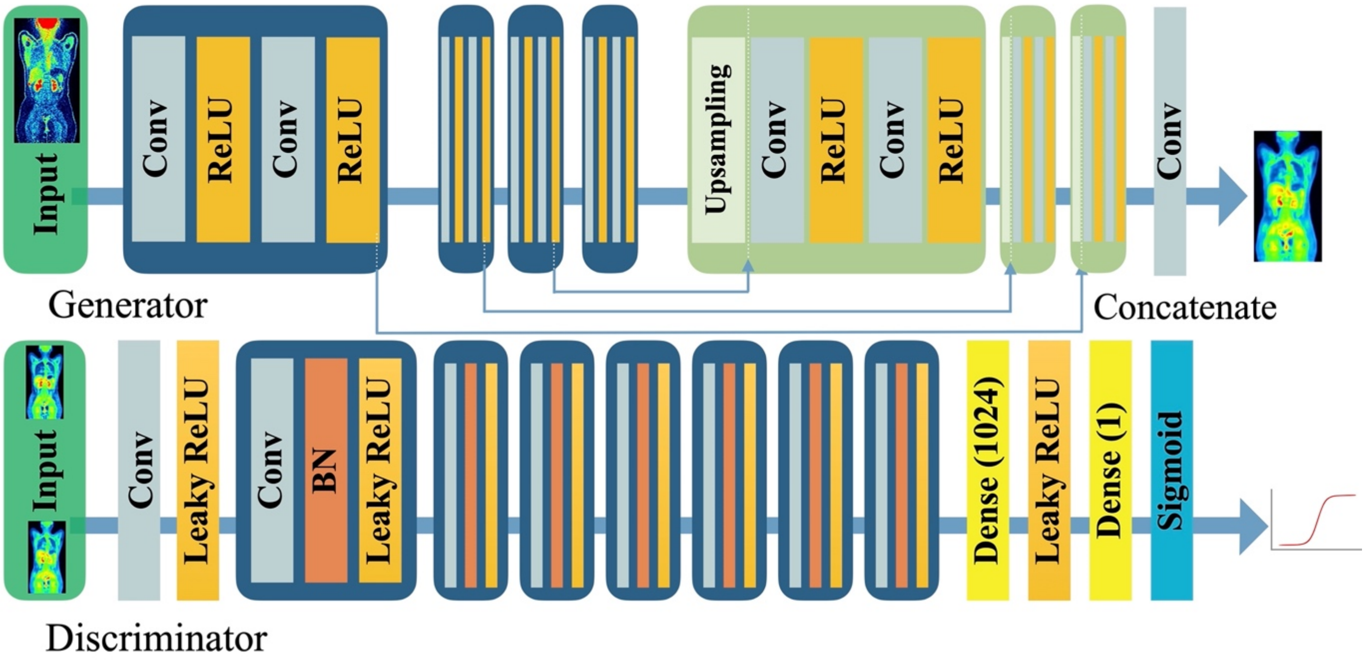

Supplementary Fig. 1 Network architecture.

Generator network: The goal of the generator is to be able to approximate the corresponding I^{ADCM} for a given $I^{NASC-PET}$. We built layers with multiple Convolution-Relu components. Specifically, the entire network constitutes 15 convolutional layers. In the encoder part which includes the first 8 convolutional layers. The number of feature maps increases from 64 in the 1st layer to 512 in the 8th layer, once every two layers, we use 3×3×3 filters and a stride of 2 every two layers. In the decoder part, we perform up-sampling with a factor of 2. Using the skip connections, the feature maps from the encoder part are copied and concatenated with the feature maps of the decoder part.

Discriminator network: The discriminator network aims to distinguish between the $\mathrm{I}^{\mathrm{S-ADCM}}$ generated by the generator model, and the real input $\mathrm{I}^{\mathrm{ADCM}}$. The discriminator takes either a ADCM or a synthesized one as input and determines whether the input is real or not. The architecture of the discriminator network contains eight convolution blocks and a fully connected block at the end. The last sigmoid activation output a probability to determine whether the input is real or synthetic. The discriminator was built with multiple convolution-batch normalizaion-Leaky Relu components. 0.2 negative slope was set for the leaky ReLu and 0.8 momentum for the batch normalization.

Equation S1

$$\min_{G}\max_{D} V(D,G) = \sum_{i=1}^{n} \log\left(\mathrm{D}_{\theta_D}(\mathrm{I}^{\mathrm{NASC-PET}}, \mathrm{I}^{\mathrm{ADCM}})\right) + \log\left(1 - \mathrm{D}_{\theta_D}\left(\mathrm{I}^{\mathrm{I}^{\mathrm{NASC-PET}}}, \mathrm{G}_{\theta_G}(\mathrm{I}^{\mathrm{I}^{\mathrm{NASC-PET}}})\right)\right)$$

In contrast to conditioning the generation of images on random noise drawn from specific distribution, this objective function takes an input of NASC-PET image. Furthermore, the adversarial loss of our model also included voxel-wise content loss alongside image-wise loss, to ensure spatial alignment of the generated ratio map with the ground truth:

Equation S2

$$L_{\text{content loss}} = \sum_{i=1}^{n} \left| I^{\text{DL-ADCM}} - I^{\text{ADCM}} \right|_2$$

Equation S3

$$\text{where } I^{\text{DL-ADCM}} = \mathrm{G}_{\theta_G}(\mathrm{I}^{\mathrm{I}^{\mathrm{NASC-PET}}})$$

Therefore, the overall objective function was defined as:

Equation S4

$$\min_{G}\max_{D} V(D,G) + \lambda V(G)$$
$$= \sum_{i=1}^{n} \log\left(\mathrm{D}_{\theta_D}(\mathrm{I}^{\mathrm{NASC-PET}}, \mathrm{I}^{\mathrm{ADCM}})\right) + \log\left(1 - \mathrm{D}_{\theta_D}\left(\mathrm{I}^{\mathrm{I}^{\mathrm{NASC-PET}}}, \mathrm{G}_{\theta_G}(\mathrm{I}^{\mathrm{I}^{\mathrm{NASC-PET}}})\right)\right)$$
$$+ \lambda \left| \mathrm{G}_{\theta_G}(\mathrm{I}^{\mathrm{I}^{\mathrm{NASC-PET}}}) - \mathrm{I}^{\mathrm{ADCM}} \right|_2$$

Training details: We employed the Adam solver [1] with a batch size of 1 and a learning rate of 0.0002. In order to facilitate efficient access to this large number of images during training, the dataset was organized into a single data object in HDF5 (Hierarchical Data Format 5). All of our experiments were implemented in TensorFlow and trained on our NVIDIA GeForce GTX 1080 Ti graphic cards. The training process ran for 100 epochs, and the weight of the content loss λ was set to 1e+4 based on experiments.

Evaluation based on Physical Metrics, Clinical and Radiomics features

Physical Metrics. To evaluate the quality of the DL ASC-PET images, we calculated and compared the following metrics: 1. Normalized root mean squared error (NRMSE); 2. Peak signal-to-noise ratio (PSNR); 3. Structural similarity index measurement (SSIM) [2]. The NRMSE is defined as:

Equation S5

$$NRMSE = \frac{\sqrt{\sum_{i=1}^{n} \frac{\left(y_{true} - y_{pred}\right)^2}{n}}}{max\left(y_{true}\right) - min\left(y_{pred}\right)}$$

where $\mathrm{y}_{\mathrm{true}}$ is the CT ASC-PET and y_{pred} is the DL ASC-PET image, and it measures the overall pixel-wise intensity deviation between these two. The PSNR is defined as:

Equation S6

$$\mathrm{PSNR} = 10\log_{10}\left(\frac{VR^2}{\left\|y_{true} - y_{pred}\right\|_2^2}\right)$$

where V is the total amounts of voxels and R represents the range of the intensity of the CT ASCT-PET image, and $\left\|y_{true} - y_{pred}\right\|_2^2$ computes the mean squared error between it and the DL ASC-PET image. The pixel-wise quantities are easily calculated and compared and have straightforward interpretations. However, they do not correspond well with the sort of errors that humans perceive, particularly blurring and smearing artifacts, and images with identical NRMSE values may appear substantially different [2]. Additional measures that more accurately reflect perceived image quality are therefore desirable.

Equation S7

$$\mathrm{SSIM}(x,y) = \frac{(2\mu_x\mu_y + C_1)(2\sigma_{xy} + C_2)}{(\mu_x^2 + \mu_y^2 + C_1)(\sigma_x^2 + \sigma_y^2 + C_2)}$$

where μ_x, μ_y are the averages of images CT ASC-PET and DL ASC-PET, and σ_x, σ_y are their standard deviations, respectively. C_1 and C_2 are two positive constants to avoid a null denominator. Theoretically, image with lower NRMSE, higher PSNR and SSIM closer to 1 represent higher synthesis quality.

Clinical and Radiomics features. Spherical VOIs were manually delineated within targeted organs (liver, kidney and heart) by two board certified nuclear medicine physicians (R.G. and C.M.) using ITK-SNAP. This was followed by standardized uptake value (SUV) discretization as well as statistical analysis. Clinical parameters and radiomics features were both included for the analysis, and we selected the mostly applied features based on references[3, 4, 5, 6, 7].

Clinical features including SUV_{mean}, SUV_{max}, total lesion metabolism (TLM), and radiomics features including root mean Squared, 90Percentile, median, joint average from gray-level co-occurrence matrix (GLCM), high gray level run emphasis from gray level Run length matrix (GLRLM), and zone percentage from gray level size zone (GLSZM). The accuracy of the clinical imaging parameters and radiomics features of the lesions within targeted organs was calculated in reference to the CT ASC-PET using mean absolute percentage error).

		Vision 450 [8]	Vision 600 [9]	uMI 780 [10]	DMI [11]
Scanner properties	NEMA Sensitivity	9.1 cps/kBq	16.4 cps/kBq	16 cps/kBq	13.3 cps/kBq
	Spatial resolution (FWHM, 1cm, radial)	3.5 mm	3.5 mm	2.9 mm	4.6 mm
	Time-of-flight resolution	213 ps	210 ps	430 ps	377 ps
Reconstruction parameters	Reconstruction algorithm	OSEM (6 iterations, 5 subsets) + TOF	OSEM (6 iterations, 5 subsets) + TOF + 3D Gaussian 5mm FWHM	OSEM (2 iterations, 20 subsets) + TOF + 3D Gaussian 2mm FWHM	OSEM (2 iterations, 17 subsets) +TOF +PSF +BPL
	Matrix size	440×440	440×440	192×192	256×256
	Gaussian filter	All-pass	5 mm	2 mm	6.4 mm

Supplementary Table. S1 Scanner properties

Results

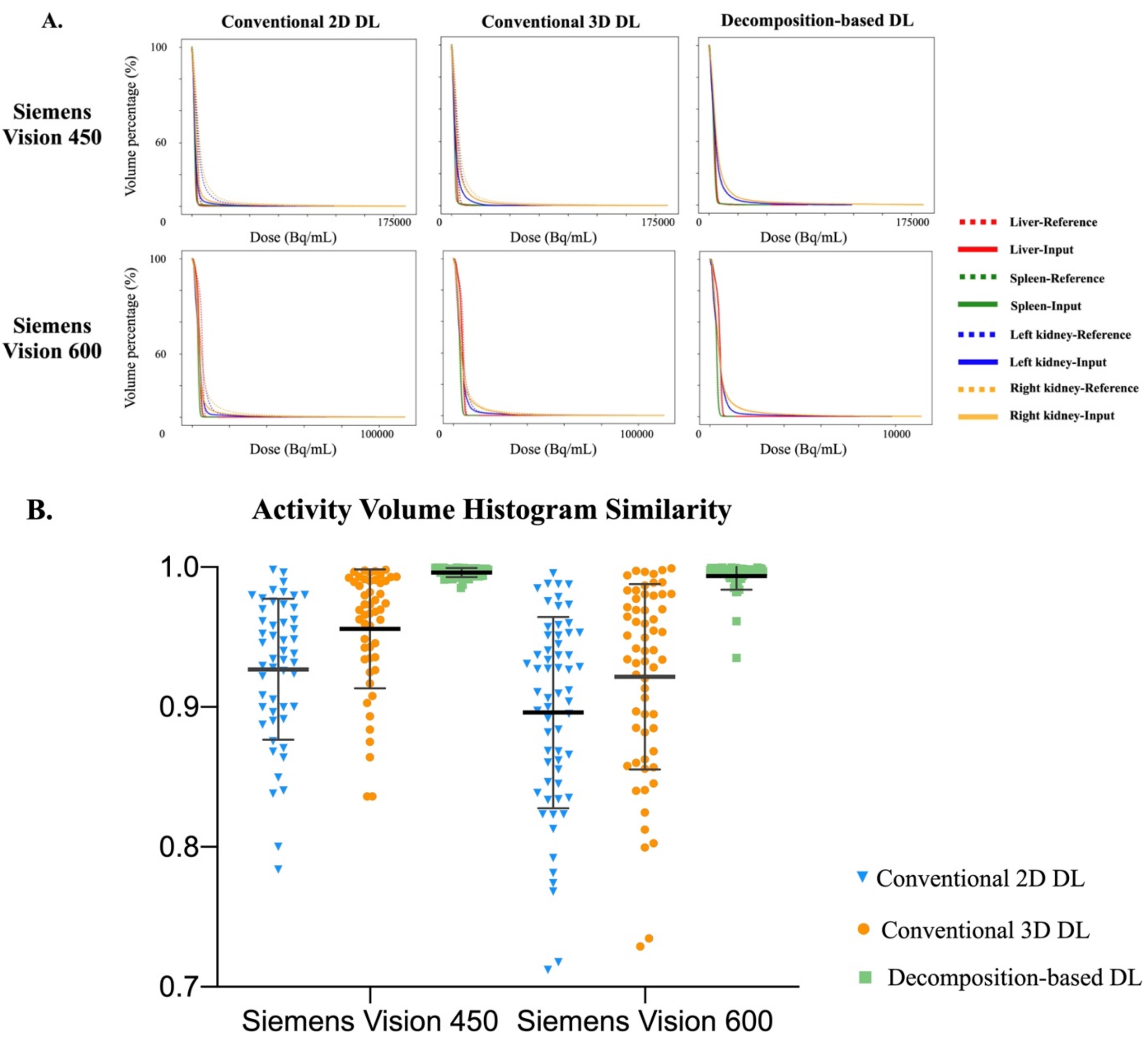

Supplementary Fig. 2 Comparison of DL approaches on activity volume histogram from Vision 450 (SH), Vision 600 (Bern) (A) and statistical similarity (B). Data are presented as mean values +/- SD. Sample size: Siemens Vision 400 (n=51), Siemens Vision 600 (n=62).

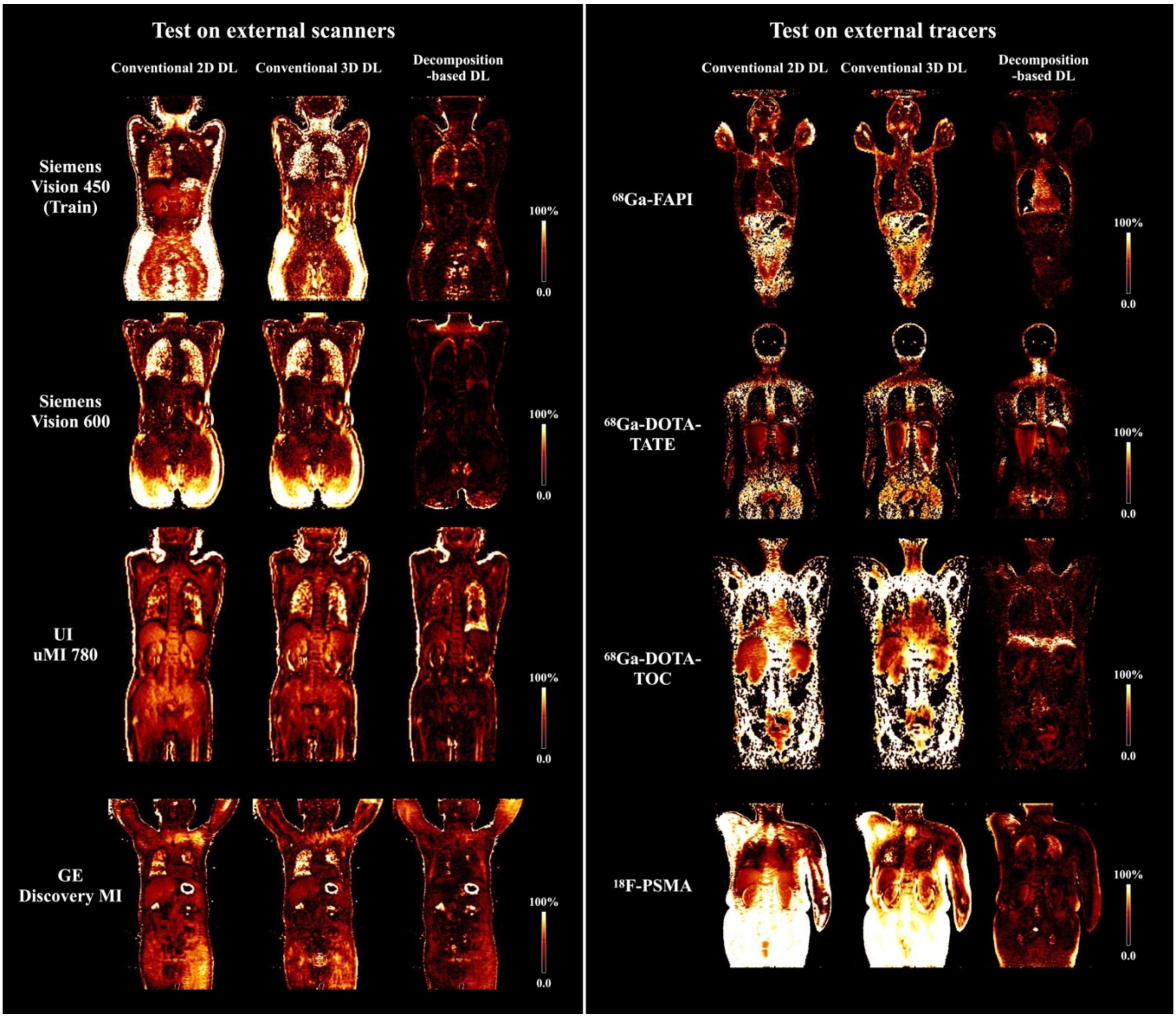

Supplementary Fig. 3 Voxel-wise absolute percentage error map of an exemplary subject depicting the difference between DL ASC-PET and reference CT ASC-PET for different scanners and tracers.

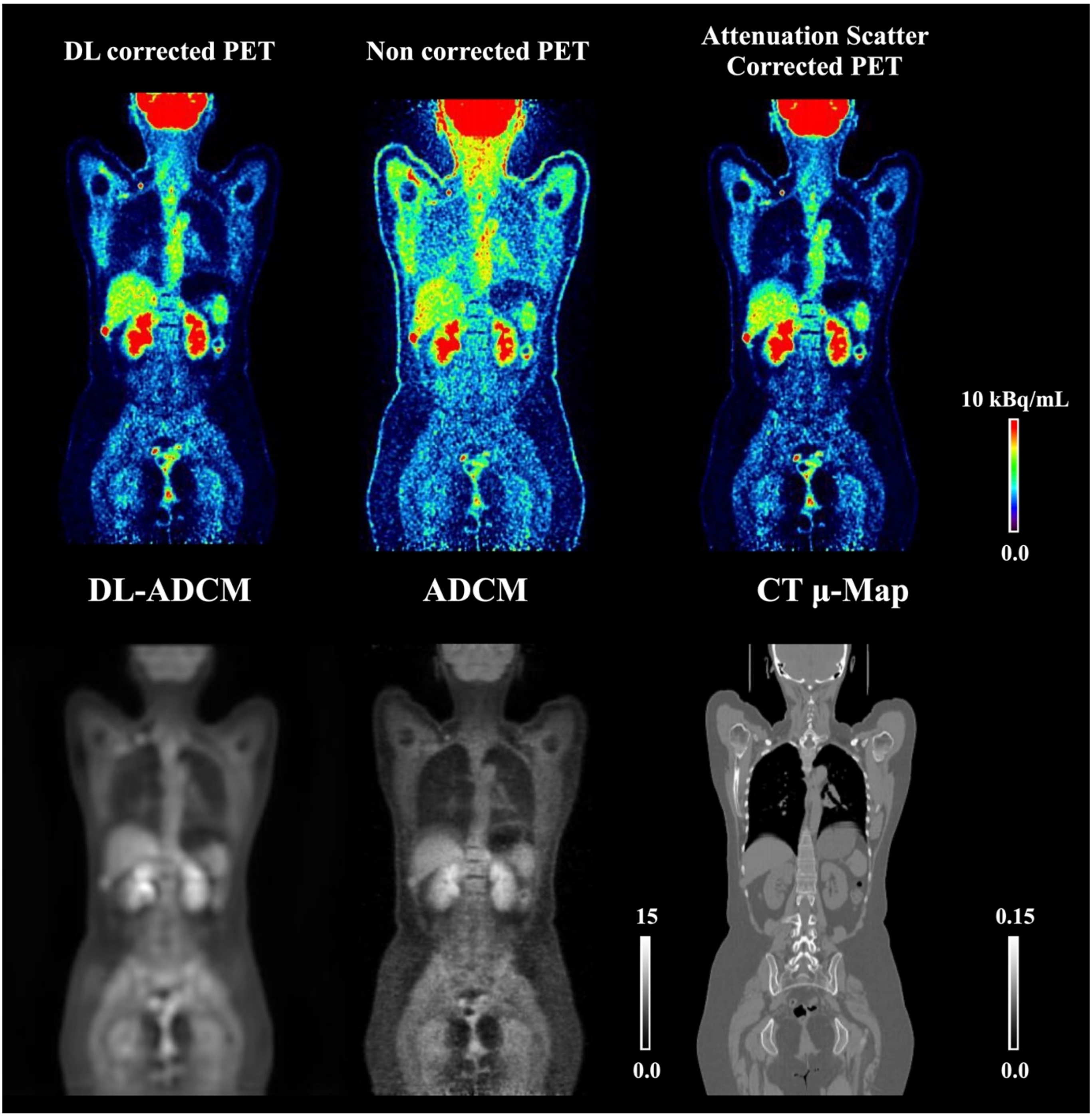

Supplementary Fig. 4 Anatomy-dependent correction map (ADCM) and deep learning-generated ADCM of an exemplary subject for different scanners and tracers.

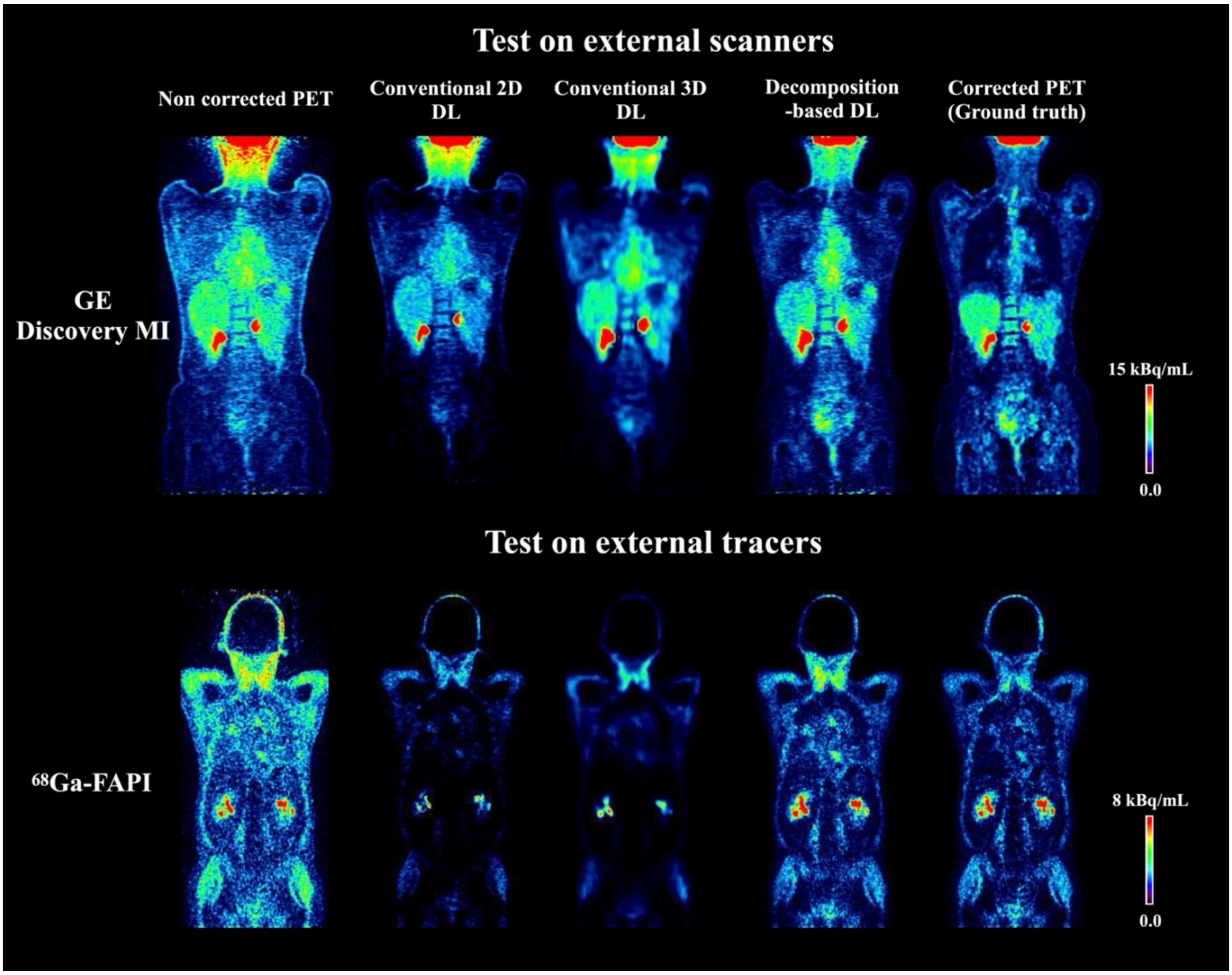

Supplementary Fig. 5 Cases showing overestimation in the neck region.

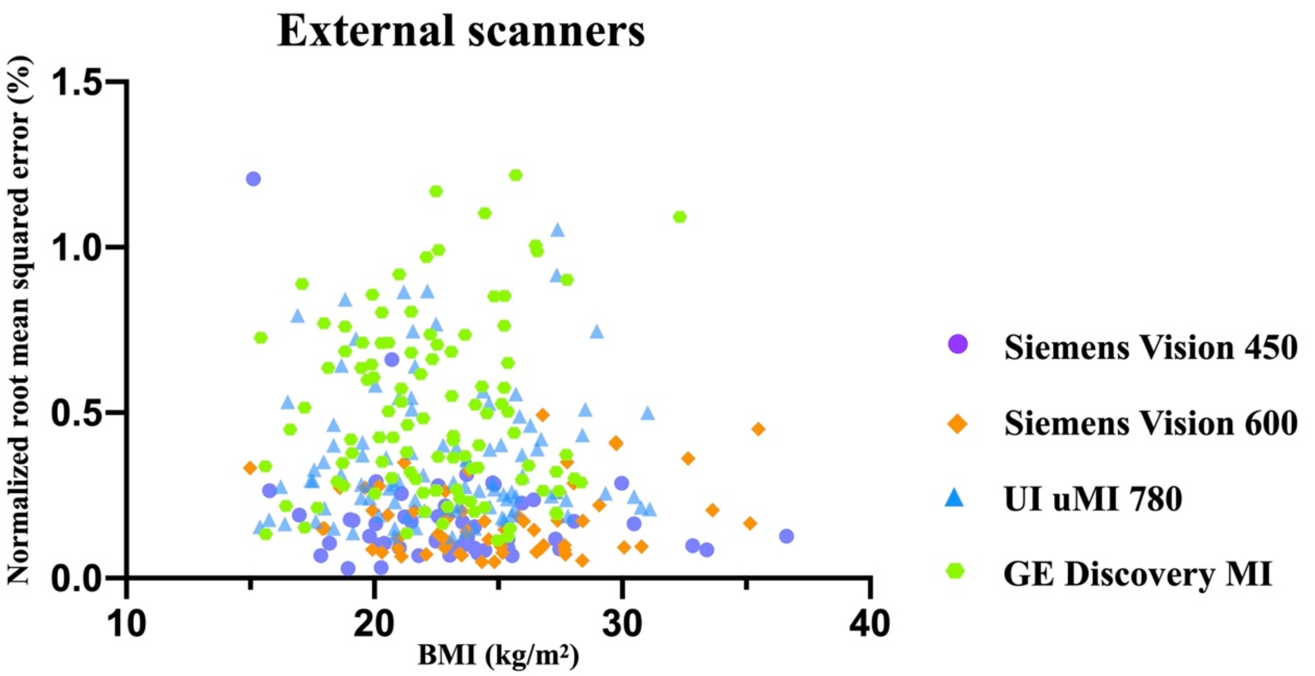

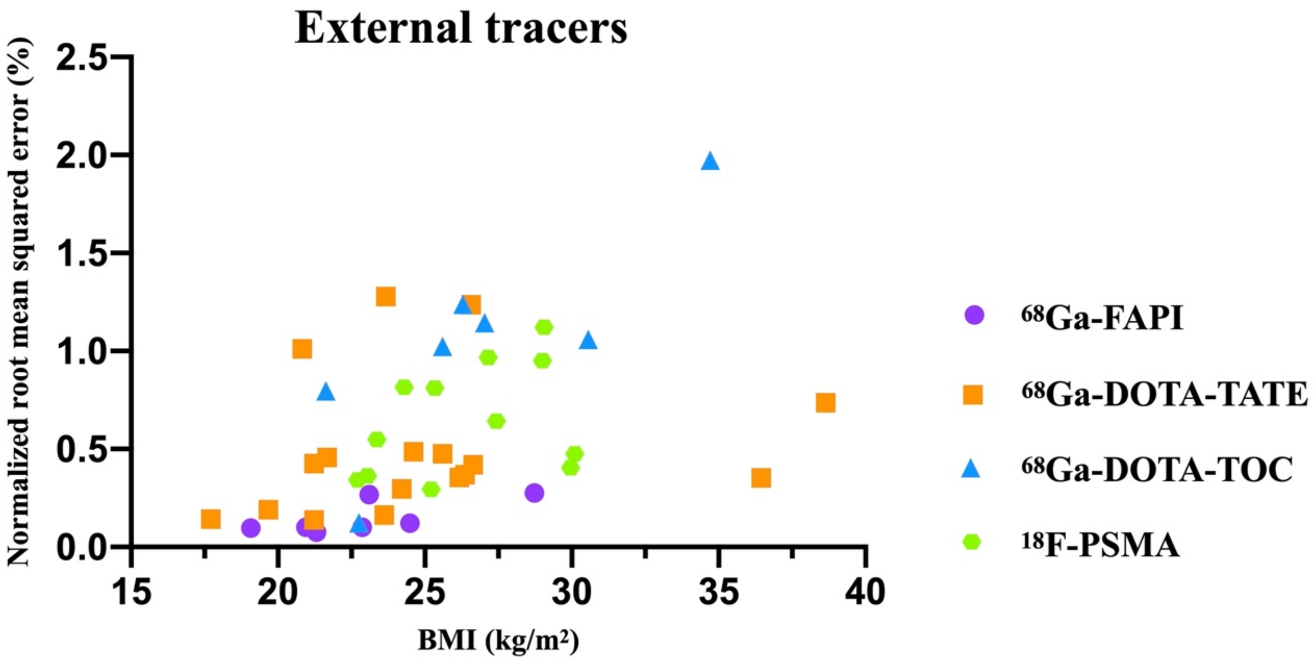

Supplementary Fig. 6 Scatter plot of relationship between body mass index and normalized root mean squared error in different scanners and tracers.

	Cross Scanner				Cross tracer			
**Source**	Vision 450 (SH)- FDG	Vision 600 (Bern)- FDG	UI uMI 780 (SH)- FDG	GE Discovery MI (SH)- FDG	Vision 450 (SH)- ^{68}Ga-FAPI	Vision 450 (SH)- ^{68}Ga-DOTA-TATE	Vision 600 (Bern)- ^{68}Ga-DOTA-TOC	Vision 600 (Bern)- ^{18}F-PSMA
**Pearson r**	-0.2671	0.1836	0.03857	0.04052	0.7152	0.1845	0.8308	0.3618
**P value (two-tailed)**	0.0582	0.1531	0.7061	0.6830	0.0708	0.4784	0.0206	0.2478

Supplementary Table. S2 Pearson test relationship between body mass index (BMI) and normalized root mean squared error (NRMSE) in different scanners and tracers. BMI is not correlated with the NRMSE.

References

1. Kingma DP, Ba J. Adam: A method for stochastic optimization. *arXiv preprint arXiv:14126980* 2014.

2. Wang Z, Bovik AC, Sheikh HR, Simoncelli EP. Image quality assessment: from error visibility to structural similarity. *IEEE transactions on image processing* 2004, **13**(4)**:** 600-612.

3. Brooks FJ, Grigsby PW. The effect of small tumor volumes on studies of intratumoral heterogeneity of tracer uptake. *J Nucl Med* 2014, **55**(1)**:** 37-42.

4. Hatt M, Majdoub M, Vallieres M, Tixier F, Le Rest CC, Groheux D*, et al.* 18F-FDG PET uptake characterization through texture analysis: investigating the complementary nature of heterogeneity and functional tumor volume in a multi-cancer site patient cohort. *J Nucl Med* 2015, **56**(1)**:** 38-44.

5. Hatt M, Tixier F, Pierce L, Kinahan PE, Le Rest CC, Visvikis D. Characterization of PET/CT images using texture analysis: the past, the present... any future? *Eur J Nucl Med Mol Imaging* 2017, **44**(1)**:** 151-165.

6. Presotto L, Bettinardi V, De Bernardi E, Belli ML, Cattaneo GM, Broggi S*, et al.* PET textural features stability and pattern discrimination power for radiomics analysis: An "ad-hoc" phantoms study. *Phys Med* 2018, **50:** 66-74.

7. Kim BH, Kim SJ, Kim K, Kim H, Kim SJ, Kim WJ*, et al.* High metabolic tumor volume and total lesion glycolysis are associated with lateral lymph node metastasis in patients with incidentally detected thyroid carcinoma. *Ann Nucl Med* 2015, **29**(8)**:** 721-729.

8. Carlier T, Ferrer L, Conti M, Bodet-Milin C, Rousseau C, Bercier Y*, et al.* From a PMT-based to a SiPM-based PET system: a study to define matched acquisition/reconstruction parameters and NEMA performance of the Biograph Vision 450. *EJNMMI physics* 2020, **7**(1)**:** 1-16.

9. van Sluis J, de Jong J, Schaar J, Noordzij W, van Snick P, Dierckx R*, et al.* Performance Characteristics of the Digital Biograph Vision PET/CT System. *J Nucl Med* 2019, **60**(7)**:** 1031-1036.

10. Hu P, Zhang Y, Yu H, Chen S, Tan H, Qi C*, et al.* Total-body (18)F-FDG PET/CT scan in oncology patients: how fast could it be? *Eur J Nucl Med Mol Imaging* 2021, **48**(8)**:** 2384-2394.

11. Chicheportiche A, Marciano R, Orevi M. Comparison of NEMA characterizations for Discovery MI and Discovery MI-DR TOF PET/CT systems at different sites and with other commercial PET/CT systems. *EJNMMI Phys* 2020, **7**(1)**:** 4.
