# Supplementary material for: Using domain knowledge for robust and generalizable deep learning-based CT-free PET attenuation and scatter correction
Source: Nat Commun. 2022 Oct 6;13:5882. doi: 10.1038/s41467-022-33562-9 (PMC9537165; doi:10.1038/s41467-022-33562-9)
Supplement: Supplementary file 3 — Reporting Summary [file 41467_2022_33562_MOESM3_ESM.pdf]

nature portfolio

Corresponding author(s): Biao Li

Last updated by author(s): Sep 13, 2022

Reporting Summary

Nature Portfolio wishes to improve the reproducibility of the work that we publish. This form provides structure for consistency and transparency in reporting. For further information on Nature Portfolio policies, see our Editorial Policies and the Editorial Policy Checklist.

Statistics

For all statistical analyses, confirm that the following items are present in the figure legend, table legend, main text, or Methods section.

n/a	Confirmed	
☐	☒	The exact sample size (*n*) for each experimental group/condition, given as a discrete number and unit of measurement
☐	☒	A statement on whether measurements were taken from distinct samples or whether the same sample was measured repeatedly
☐	☒	The statistical test(s) used AND whether they are one- or two-sided<br>*Only common tests should be described solely by name; describe more complex techniques in the Methods section.*
☒	☐	A description of all covariates tested
☒	☐	A description of any assumptions or corrections, such as tests of normality and adjustment for multiple comparisons
☒	☐	A full description of the statistical parameters including central tendency (e.g. means) or other basic estimates (e.g. regression coefficient) AND variation (e.g. standard deviation) or associated estimates of uncertainty (e.g. confidence intervals)
☐	☒	For null hypothesis testing, the test statistic (e.g. *F*, *t*, *r*) with confidence intervals, effect sizes, degrees of freedom and *P* value noted<br>*Give P values as exact values whenever suitable.*
☒	☐	For Bayesian analysis, information on the choice of priors and Markov chain Monte Carlo settings
☒	☐	For hierarchical and complex designs, identification of the appropriate level for tests and full reporting of outcomes
☒	☐	Estimates of effect sizes (e.g. Cohen's *d*, Pearson's *r*), indicating how they were calculated

Our web collection on statistics for biologists contains articles on many of the points above.

Software and code

Policy information about availability of computer code

Data collection	The data were collected directly from all commercial PET scanners, including Siemens Biograph Vision 450 and 600 (Siemens Healthineers; Software: Biograph Vision PETsyngo; Version: VG76B), United Imaging uMI 780 (United Imaging; software: uExceed; Version:R001.10.0.1138038) and Discovery MI (General Electric Healthcare, software: Advantage Workstation; version AW 4.7) .
Data analysis	Data analysis was performed with customized codes. Following the comments from the editor, we marked it clearly with version XXX. It is now publicly available on GitHub with a link in this paper.

For manuscripts utilizing custom algorithms or software that are central to the research but not yet described in published literature, software must be made available to editors and reviewers. We strongly encourage code deposition in a community repository (e.g. GitHub). See the Nature Portfolio guidelines for submitting code & software for further information.

Data

Policy information about availability of data

All manuscripts must include a data availability statement. This statement should provide the following information, where applicable:

- Accession codes, unique identifiers, or web links for publicly available datasets
- A description of any restrictions on data availability
- For clinical datasets or third party data, please ensure that the statement adheres to our policy

The data that support the findings of this study can be available after data transfer agreement, which can be requested to the corresponding author (BL).

Human research participants

Policy information about studies involving human research participants and Sex and Gender in Research.

Reporting on sex and gender	There are 446 males and 383 females included in this study.
Population characteristics	We described the information of population within Table 1, including the injected dose, post-injection time, gender, age and weight.
Recruitment	This is a retrospective study, 8 cohorts with 829 subjects from two centers scanned with PET/CT were enrolled.
Ethics oversight	The study complies with all relevant ethical regulations of the respective local ethics committees in Switzerland (Swiss Association of Research Ethics Committees) and China (Ruijin Hospital Ethics Committee Shanghai Jiao Tong University School of Medicine).

Note that full information on the approval of the study protocol must also be provided in the manuscript.

Field-specific reporting

Please select the one below that is the best fit for your research. If you are not sure, read the appropriate sections before making your selection.

[x] Life sciences [ ] Behavioural & social sciences [ ] Ecological, evolutionary & environmental sciences

For a reference copy of the document with all sections, see nature.com/documents/nr-reporting-summary-flat.pdf

Life sciences study design

All studies must disclose on these points even when the disclosure is negative.

Sample size	The small size of medical datasets continues to be an issue in obtaining satisfactory deep learning model, so we included the largest amount of available data to develop and test our deep learning techniques
Data exclusions	No data were excluded.
Replication	The experiments were repeated for more than five times independently, and all achieved similar level of performance.
Randomization	The allocation of sample for the development was completely randomized. The test dataset included the rest of all data available, hence there was no need for randomization. Covariate is not relevant to our study.
Blinding	The investigator was blind to the collection and allocation during data collection, and presented results for all subjects from the test dataset provided, so there's no need for blinding.

Reporting for specific materials, systems and methods

We require information from authors about some types of materials, experimental systems and methods used in many studies. Here, indicate whether each material, system or method listed is relevant to your study. If you are not sure if a list item applies to your research, read the appropriate section before selecting a response.

Materials & experimental systems

n/a	Involved in the study	
[x]	[ ]	Antibodies
[x]	[ ]	Eukaryotic cell lines
[x]	[ ]	Palaeontology and archaeology
[x]	[ ]	Animals and other organisms
[x]	[ ]	Clinical data
[x]	[ ]	Dual use research of concern

Methods

n/a	Involved in the study	
[x]	[ ]	ChIP-seq
[x]	[ ]	Flow cytometry
[x]	[ ]	MRI-based neuroimaging
